# Interfering with Glycolysis Causes Sir2-Dependent Hyper-Recombination of *Saccharomyces cerevisiae* Plasmids

**DOI:** 10.1371/journal.pone.0005376

**Published:** 2009-04-24

**Authors:** Markus Ralser, Ute Zeidler, Hans Lehrach

**Affiliations:** 1 Max Planck Institute for Molecular Genetics, Berlin, Germany; 2 Department of Cell Biology, University of Salzburg, Salzburg, Austria; University of Texas-Houston Medical School, United States of America

## Abstract

Glyceraldehyde-3-phosphate dehydrogenase (GAPDH) is a key metabolic regulator implicated in a variety of cellular processes. It functions as a glycolytic enzyme, a protein kinase, and a metabolic switch under oxidative stress. Its enzymatic inactivation causes a major shift in the primary carbohydrate flux. Furthermore, the protein is implicated in regulating transcription, ER-to-Golgi transport, and apoptosis. We found that *Saccharomyces cerevisiae* cells null for all GAPDH paralogues (Tdh1, Tdh2, and Tdh3) survived the counter-selection of a GAPDH–encoding plasmid when the NAD^+^ metabolizing deacetylase Sir2 was overexpressed. This phenotype required a fully functional copy of *SIR2* and resulted from hyper-recombination between *S. cerevisiae* plasmids. In the wild-type background, GAPDH overexpression increased the plasmid recombination rate in a growth-condition dependent manner. We conclude that GAPDH influences yeast episome stability via Sir2 and propose a model for the interplay of Sir2, GAPDH, and the glycolytic flux.

## Introduction

Glyceraldehyde-3-phosphate dehydrogenase (GAPDH) is a central metabolic regulator named for its enzymatic conversion of glyceraldehyde-3-phosphate into 1,3-bisphosphoglycerate in the sixth step of glycolysis [1]. The glycolytic activity of GAPDH can be modified by a variety of conditions. For instance, GAPDH is redox sensitive and is inactivated in oxidant-treated bacteria, yeast, nematode, mammalian, and plant cells[2]–[8]. Moreover, alterations in GAPDH activity have been detected in a variety of disorders, including cancer, diabetes, Huntington's disease, and Alzheimer's disease [9]–[11].

Oxidation of cysteine residues in the active site of GAPDH replenishes its activity under oxidative stress [2], [3]. Several other modifications, including S-nitrosylation, S-thiolation, carbonylation, and ADP-ribosylation, have also been reported to contribute to or accompany this process [4]–[7], [12], [13]. Interestingly, the inactivation of GAPDH causes a re-direction of metabolic flux from glycolysis into the pentose phosphate pathway [14]. This metabolic re-configuration results in the recycling of NADPH, a major redox cofactor in the antioxidant machinery and the source of redox power for glutathione recycling [14], [15].

In addition to being an important catabolic enzyme, GAPDH is a key regulatory modulator in a variety of processes. For instance, GAPDH can act as a protein kinase by phosphorylating the long intracellular loop of the GABA(A) receptor alpha 1 subunit, thereby regulating synaptic transmission in neurons [16], and influences the viral lifecycle by phosphorylating the hepatitis B virus core protein [17]. GAPDH can also contribute to the initiation of apoptosis (reviewed in [13]), for instance, by binding to the ubiquitin ligase *Siah1* in response to cellular stress and translocating into the nucleus. There, the complex targets nuclear proteins for degradation [18]. GAPDH also participates in ER vesicle-to-Golgi transport. Upon activation via tyrosine phosphorylation by Src, GAPDH is recruited by Rab2 to the vesicular-tubular clusters of the ER, where it helps to form *COP1* vesicles [19].

Finally, GAPDH can activate transcription. GAPDH and lactate dehydrogenase are part of the *OCA-S* transcriptional coactivator complex that links the metabolic state to gene transcription [20]. Moreover, the yeast GAPDH paralogue Tdh3 interacts genetically with Sir2 [21], a member of the Sirtuin family of proteins, which function as NAD^+^-dependent protein deacetylases. Sirtuins have a well-established role in deacetylating histones and are essential for gene silencing and chromatin stability [22]. Like GAPDH, Sirtuins are conserved metabolic regulators [23] and play a still controversial role in the cellular aging process [24].

Evidence for an interaction between GAPDH and Sir2 include data from a *S. cerevisiae* screen for multicopy suppressors of lethality caused by *GAL1*-promoter driven overexpression of Sir2 [21]. In addition to histone 4, two ribosomal proteins, and the sphinganine C4-hydroxylase Sur2, overexpression of the predominant yeast GAPDH paralogue, Tdh3, suppressed Sir2-induced lethality. Moreover, a large scale study revealed that Sir2 and Tdh3 were present in a protein complex purified by a TAP-tagging strategy [25]. These results suggest a close relationship between GAPDH and Sir2, although the details of their genetic and biochemical interactions are not understood.

## Results and Discussion

We generated a yeast model for studying different GAPDH species by deleting the genes encoding the three GAPDH isozymes (Tdh1, Tdh2 and Tdh3) in a commonly used *S. cerevisiae* strain (BY4741). To prevent the synthetic lethal phenotype of Δ*tdh1*Δ*tdh2*Δ*tdh3* triple deletion mutants [26], we introduced a counter-selectable plasmid carrying the *E. coli* GAPDH paralogue *Eco*GAP (79% amino acid similarity to Tdh3 by Blossum62) into the parent strain before deletion of the genomic loci.

We used this strain to study GAPDH activity by performing classic 5′fluoroorotic acid (5′FOA) plasmid shuffle assays, in which a second plasmid carrying a *HIS3* marker and the gene to be studied were introduced into cells. Then, clones containing both the *URA3* and the *HIS3* plasmid were selected on synthetic media lacking histidine and uracil (SC^-HIS-URA^), grown overnight, and spotted in a five-fold dilution series on synthetic complete media with or without 0.15% 5′FOA. Only yeast cells deficient for uracil synthesis (cells that have lost the GAPDH-encoding *URA3* plasmid) are able to grow on the 5′FOA containing media; thus, only cells in which the *HIS3* plasmid compensates for the loss of the GAPDH plasmid are viable.

**Figure 1:**
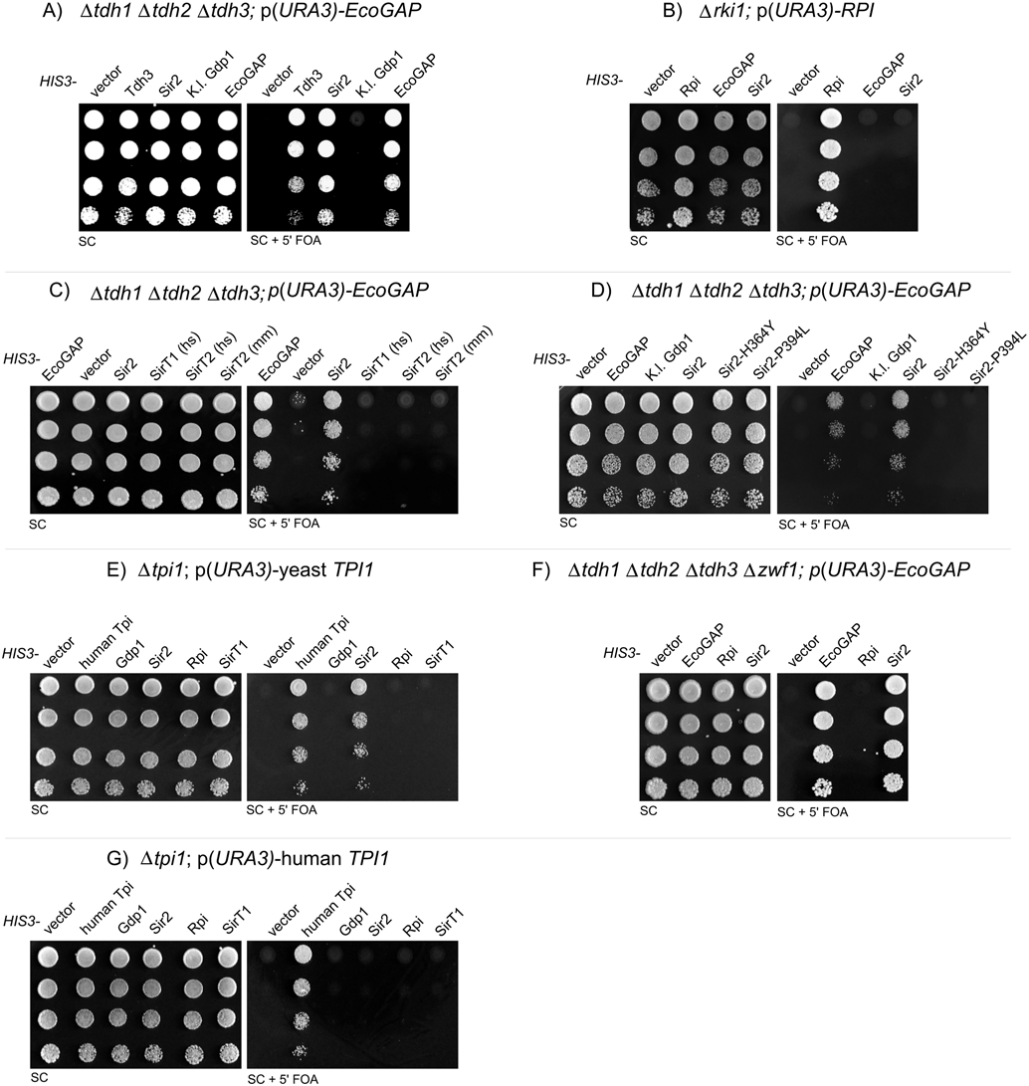
Δ*tdh1*Δ*tdh2*Δ*tdh3* yeast survives counter-selection of a GAPDH-encoding plasmid when Sir2 is overexpressed. (A) The Δ*tdh1*Δ*tdh2*Δ*tdh3* strain MR173 containing the plasmid p(*URA3*)-*Eco*GAP was transformed with the indicated *HIS3* plasmids. Transformants were selected, grown overnight, and spotted in a five-fold serial dilution on SC media containing 5′FOA for counter-selection of the *URA3* plasmid or on SC media as control. Plates were then incubated for 3 days at 30°C. (B) Similar experiment as described in (A), but using Δ*rki1* yeast expressing the human *RKI1* orthologue (Rpi1) from the *URA3* plasmid. (C) Δ*tdh1*Δ*tdh2*Δ*tdh3* yeast overexpressing Sir2 or its mammalian homologues, human SirT1, human SirT2, and mouse SirT2, were processed as in (A). (D) Similar to (C), but overexpressing the mutant proteins Sir2^H364Y^ and Sir2^P394L^. (E) The yeast strain MR110, in which chromosomal *TPI1* is deleted and yeast *TPI1* is expressed from an *URA3* episome, was transformed with the indicated *HIS3* plasmids and processed as described above. (F) Similar experiment using the quadruple deletion strain Δ*tdh1*Δ*tdh2*Δ*tdh3*Δ*zwf1*. (G) Similar to (E), but using yeast strain MR101, which is isogenic to MR110, but expresses human instead of yeast Tpi1 from the *URA3* episome.

A typical experiment is illustrated in Figure 1A. Yeast cells expressing Tdh3 or *Eco*GAP from the *HIS3* episome were viable on 5′FOA media. In turn, yeast cells harboring the empty *HIS3* plasmid or a plasmid encoding for *Kluyveromyces lactis* Gdp1, were not viable. This demonstrates that ectopic expression of GAPDH isozymes can rescue for the loss of the chromosomally encoded GAPDH genes. *Kluyveromyces lactis* Gdp1 is highly homologous to Tdh3 (76% similarity), and both enzymes catalyze the conversion of glyceraldehyde-3-phosphate to 1,3-bisphosphoglycerate; the only difference is that the redox cofactor: Gdp1 depends on NADP(H) [27]. The fact that Gdp1 expression does not compensate for the loss of GAPDH underscores the high specificity of the assay.

Surprisingly, we discovered that Δ*tdh1*Δ*tdh2*Δ*tdh3* cells transformed with a plasmid overexpressing Sir2 also grew on 5′FOA media (Figure 1A), indicating functional rescue of GAPDH by Sir2. However, Sir2 has been extensively studied at the molecular level, and there has been no indication that Sir2 catalyzes NAD(H)-dependent oxidative phosphorylation of glyceraldehyde-3-phosphate. To exclude the possibility that this result was an artifact of the 5′FOA assay, for instance through a Sir2-dependent increase in the 5′FOA resistance of the yeast, we plated the counter-selected cells on SC^-URA^ and SC^-HIS^ plates. We found that they were viable on SC^-HIS^ but unable to grow on SC^-URA^, confirming that the *URA3* plasmid was indeed counter-selected (data not shown).

Next, to test for the specificity of the Sir2-GAPDH interaction, we generated a yeast strain in which an unrelated enzyme, the ribulose-5-ketol-isomerase *RKI1*, was deleted and tested for rescue by Sir2. Like GAPDH, Rki1 is essential for cytoplasmic carbohydrate metabolism and is highly conserved. Rki1 is encoded by a single yeast gene and has no other paralogues in *S. cerevisiae*; the *Δrki1* strain was kept viable by expression of the human *RKI1* orthologue Rpi1 from an *URA3* plasmid. We performed a counter-selection assay with this strain, as illustrated in Figure 1B. Yeast cells carrying the *HIS3* plasmid encoding Rpi1 were viable on 5′FOA media, whereas cells carrying the empty vector were not, confirming that human Rpi compensates for the loss of its yeast orthologue. However, yeast cells with plasmids encoding *Eco*GAP or Sir2 did not grow on 5′FOA media, indicating that *Eco*GAP and Sir2 cannot rescue cells with the *RPI1* deletion. Thus, the rescue of Δ*tdh1*Δ*tdh2*Δ*tdh3* cells by Sir2 overexpression was specific.

GAPDH and Sir2 are both dependent on the metabolic cofactor NAD^+^. Whereas GAPDH reduces NAD^+^ to NADH during glycolysis, Sir2 transfers an acetyl group to the ADP-ribose part of NAD^+^, forming O-acetyl-ADP-ribose. To determine whether the rescue of Δ*tdh1*Δ*tdh2*Δ*tdh3* cells by Sir2 overexpression is a direct or indirect consequence of this metabolic relationship, we cloned two mammalian cytoplasmic Sirtuins, SirT1 and SirT2, into a *HIS3*-containing centromeric plasmid with a *TEF1* promoter. SirT1 is the direct mammalian orthologue of Sir2, whereas SirT2 targets other acetylated substrates, but is nonetheless a NAD^+^ dependent, O-acetyl-ADP-ribose-forming deacetylase. As illustrated in Figure 1C, overexpression of human SirT1, human SirT2, and mouse SirT2 failed to rescue Δ*tdh1*Δ*tdh2*Δ*tdh3* cells after counter-selection for the GAPDH-encoding plasmid. Thus, overexpression of other NAD^+^-dependent deacteylases was not sufficient to promote survival of Δ*tdh1*Δ*tdh2*Δ*tdh3* cells.

We next used site-directed PCR mutagenesis to perform structure-function studies of Sir2 in Δ*tdh1*Δ*tdh2*Δ*tdh3* yeast cells overexpressing *EcoGAP*. One mutation substitutes a tyrosine for a phylogenetically invariant histidine residue, thereby abolishing the deacetylase activity of Sir2 (Sir2^H364Y^) [28]. As illustrated in Figure 1D, Δ*tdh1*Δ*tdh2*Δ*tdh3* cells expressing Sir2^H364Y^ are unable to grow on media containing 5′FOA. These results indicate that the enzymatic activity of overexpressed Sir2 is essential for its ability to rescue cells lacking GAPDH. We also generated another mutant, Sir2^P394L^, which has normal catalytic activity but is deficient in homotrimerization [29]. Sir2^P394L^ is not able to rescue the GAPDH-deficient cells (Figure 1D). Together, these results show that the NAD^+^-dependent deacetylase activity of Sir2 is necessary but not sufficient to rescue Δ*tdh1*Δ*tdh2*Δ*tdh3* cells.

GAPDH acts as an enzymatic metabolic switch; once inactivated, the cytoplasmic carbohydrate flux re-routes from glycolysis to the pentose phosphate pathway [14]. This alters the redox state of the cell and is required for cellular survival under oxidative stress [4], [14], [30]. Inactivation of Triose phosphate isomerase (Tpi), the enzyme that catalyzes the glycolytic step preceding the one catalyzed by GAPDH, has similar metabolic consequences as the inactivation of GAPDH. Hence, *TPI1* mutants can be used to distinguish between direct (enzymatic or other direct activities of GAPDH) and indirect (metabolic alterations in the carbohydrate flux) consequences of GAPDH inactivation.

To determine whether Sir2 overexpression has similar effects on yeast cells ectopically expressing Tpi1, we tested a *Δtpi1* yeast strain (MR110) that expresses *S. cerevisiae* Tpi1 from a *URA3* plasmid [31]. As illustrated in Figure 1E, neither the empty vector, nor *K. lactis* Gdp1, Rpi1, or SirT1 overexpression allowed Δ*tpi1* cells to grow on 5′FOA. As expected [31], human Tpi1 complemented for the growth phenotype of Δ*tpi1*. However, also overexpression of Sir2 allowed the Δ*tpi1* cells to grow on 5′FOA-containing media. These results indicate that rescue of GAPDH-deficient cells by Sir2 overexpression depends on the glycolytic and not the non-glycolytic functions of GAPDH. Moreover, this experiment demonstrates that Sir2 does not compensate for GAPDH by acting as a glyceraldehyde-3-phosphate dehydrogenase; such an enzymatic activity would not rescue yeast cells deleted for *TPI1* but wild-type for GAPDH.

Glycolysis is interconnected with the pentose phosphate pathway (PPP). Although glucose equivalents can be fully metabolized in the PPP, use of this pathway alone does not support cellular survival. For instance, deletion of the phosphoglucose-isomerase gene *PGI1* blocks the entry of sugar phosphates into glycolysis and is lethal under standard conditions, even though glucose equivalents can still be metabolized in the PPP [32]. However, *PGI1* mutant yeast are viable when they overexpress *Kluyveromyces lactis* Gdp1 or the glutamate dehydrogenase Gdh2 [27], [32]. To test whether Sir2 overexpression results in a metabolic reconfiguration that allows cellular survival without glycolysis, we generated a quadruple-mutant in which all three GAPDH genes and the Glucose-6-phosphate dehydrogenase *ZWF1* are deleted. Deletion of the Zwf1 enzyme prevents the shunting of glucose equivalents from glycolysis into the PPP. As illustrated in Figure 1F, Δ*tdh1*Δ*tdh2*Δ*tdh3*Δ*zwf1* yeast overexpressing Sir2 showed the same amount of growth on 5′FOA media as the isogenic *ZWF1*-wild-type strain. Thus, Sir2 overexpression does not promote survival of cells lacking GAPDH by redirection the metabolic flux through the PPP.

We next performed similar experiments in yeast strains with reduced Tpi activity. Human Tpi has 68% amino acid similarity to yeast Tpi (Blossum62) and complements for the growth phenotypes of *Δtpi1* cells [14]. Surprisingly, in strains expressing human Tpi, Sir2 overexpression does not permit cell growth on 5′FOA media (Figure 1G). We also performed the experiment in a yeast strain expressing a Tpi mutant, Tpi^Ile170Val^, that has only 30% activity compared to wild-type human Tpi [31], and obtained similar results (data not shown). First, this finding indicates that human Tpi is not able to complement to 100% for yeast Tpi. For instance the binding affinities of regulatory Tpi interactors could be different. This is consistent with our previous findings showing that yeast cells expressing human Tpi have only 70% overall Tpi activity compared to a wild-type strain, and display an increased resistance against the oxidant diamide [14], [31].

Still, this result is very surprising, because the strains we used were direct descendants of the same parent strain (MR100) and should therefore not differ after counterselection of the Tpi encoding *URA3* plasmid. Possible explanations for this unforeseen result include epigenetic differences between the two strains or the presence of the different Tpi isoforms in the cell even after counter-selection of the *URA3* plasmid.

**Figure 2:**
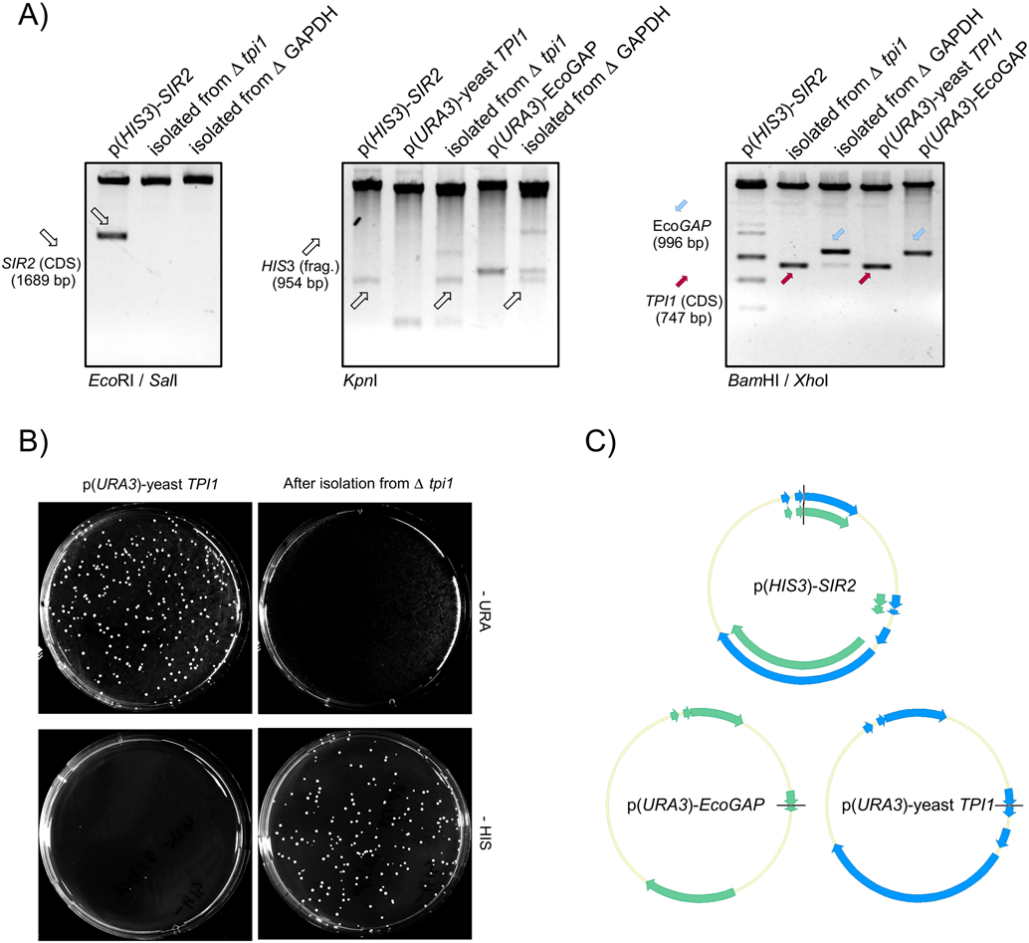
Deregulation of the glycolysis/Sir2 equilibrium causes plasmid recombination. (A) Plasmids isolated from 5′FOA counter-selected, Sir2-overexpressing Δ*tpi1* and Δ*tdh1*Δ*tdh2*Δ*tdh3* (Δ GAPDH) cells were amplified in *E. coli* and digested with *Eco*RI/*Sal*I (left panel), *Kpn*I (middle panel) or *Bam*HI/*Xho*I (right panel) and compared with equivalently-treated p(*HIS3*)-*SIR2*, p(*URA3*)-yeast *TPI1*, or p(*URA3*)-EcoGAP, respectively. (B) p(*URA3*)-*TPI1* and the Tpi-encoding plasmid isolated from 5′FOA counter-selected, Sir2-overexpressing Δ*tpi1* yeast were transformed into BY4741, plated on SC^-HIS^ and SC^-URA^ media and incubated at 30°C. (C) Plasmid sequences of p(*URA3*)-*Eco*GAP and p(*URA3*)-yeast Tpi1 were aligned to the sequence of p(*HIS3*)-*SIR2*. Identical regions between p(*URA3*)-*Eco*GAP and p(*HIS3*)-*SIR2* are highlighted in green, regions shared between p(*URA3*)-yeast *TPI1* and p(*HIS3*)-*SIR2* in blue.

Therefore, we re-isolated *HIS3* plasmids from counter-selected Δ*tdh1*Δ*tdh2*Δ*tdh3* and Δ*tpi1* yeast. The plasmids were re-transformed into *E. coli* for amplification and subsequently analyzed by restriction digest with *Eco*RI/*Sal*I, because the original *HIS3* plasmid was constructed by ligating the Sir2 coding sequence into the *Eco*RI/*Sal*I sites of the vector p413TEF. As illustrated in Figure 2A (left panel), this produced the expected 1689 bp Sir2-containing band. Surprisingly, however, this DNA fragment was not detectable in the *HIS3* plasmids that were re-isolated from the counter-selected yeast.

We next digested the plasmids with *Kpn*I, which produces a 954 bp band due to an internal *Kpn*I site in the *HIS3* marker (Figure 2A, middle panel). This band was found in digests of the original Sir2 vector and plasmids re-isolated from counter-selected Δ*tdh1*Δ*tdh2*Δ*tdh3* and Δ*tpi1* cells, but not in digests of the original *URA3* plasmids encoding *Eco*GAP or Tpi1. Hence, the plasmid isolated from the counter-selected yeast strains was neither the original plasmid harboring Sir2, nor the plasmid used to express GAPDH or Tpi.

Other *Kpn*I restriction fragments detectable in p(*URA3*)-*Eco*GAP or p(*URA3*)-*TPI1* were observed from plasmids isolated from Δ*tdh1*Δ*tdh2*Δ*tdh3* or Δ*tpi1* yeast. This suggested that the re-isolated plasmids were recombined hybrids of the original *URA3* and *HIS3* vectors. To test this, we performed a third digest using *Bam*HI and *Xho*I. This produced a 996 bp fragment corresponding to the *Eco*GAP coding sequence from p(*URA3*)-*Eco*GAP and a 747 bp fragment corresponding to the *TPI1* coding sequence from p(*URA3*)-*TPI1*, but produced multiple fragments from the original Sir2-encoding plasmid (Figure 2A, right panel). Indeed, the plasmid isolated from the Δ*tdh1*Δ*tdh2*Δ*tdh3* strain produced a *Bam*HI/*Xho*I fragment corresponding to the *Eco*GAP coding sequence, whereas the Δ*tpi1* strain produced a fragment corresponding to yeast Tpi1. Thus, the plasmid isolated from the Δ*tdh1*Δ*tdh2*Δ*tdh3* strain contained a *HIS3* marker and the *Eco*GAP coding sequence. Similarly, the plasmid isolated from Δ*tpi1* yeast encoded *HIS3* and Tpi1. Hence, the new plasmids were recombined hybrids of the two original plasmids.

The original plasmids shared a number of sequence features. In Figure 2C, the plasmid regions with 100% identity (e.g.the ampicillin-resistance gene, the *E. coli* replication-origins, and the *ARS* sequences) are highlighted; more than 30% of the sequence was found in all plasmids. The high homology of the shared plasmid features and the fact that all extracted plasmids showed a similar restriction pattern suggests that homologous recombination between vectors caused the observed phenomena.

To further dissect the putative recombination events, we re-transformed the original and re-isolated Tpi1-encoding plasmids into the yeast strain BY4741. As illustrated in Figure 2B, yeast transformed with p(*URA3*)-yeast *TPI1* grew on SC^–URA^ plates, but not on SC^–HIS^ plates. The Tpi encoding plasmid re-isolated from the Δ*tpi1* strain resulted in colonies on SC^–HIS^, but not on SC^–URA^. Identical results were obtained with the plasmids encoding *Eco*GAP. Therefore, the viability of the counter-selected Δ*tdh1*Δ*tdh2*Δ*tdh3* and *Δtpi1* cells can be explained by plasmid recombination; after expressing Sir2, these strains contained a new *HIS3* plasmid encoding either GAPDH or Tpi.

Other laboratories have found that the lack of Sir2 results in hyper-recombination of rDNA repeat units [33], [34]. The most obvious difference between our studies and theirs is that our experiments were performed in yeast strains not wild-type for glycolysis. Consequently, we extended our investigations by studying plasmid recombination in a wild-type background. For this, we developed a plasmid-recombination assay using the *E. coli* ß-galactosidase gene *lacZ* as a reporter. We PCR-amplified 5′ and 3′ fragments of the *lacZ* gene from *E. coli* genomic DNA (strain GM2929) that overlapped by 564 bp. The 5′ fragment was cloned into the 2 µ *URA3* expression vector p426GPD under the control of the constitutive *GPD1* promoter, and the 3′ fragment was cloned into the *LEU2* containing 2 µ vector pRS425. Neither plasmid alone is able to produce a functional *lacZ* enzyme, but upon plasmid recombination, a functional *lacZ* gene is reconstituted and yeast cells turn blue in an X-GAL assay (Figure 2D).

To test the influence of GAPDH and Sir2 overexpression on plasmid recombination, we transformed both *lacZ* reporter plasmids into the yeast strain BY4741. Single colonies were selected and transformed with a third, *HIS3*-containing plasmid encoding Sir2 or GAPDH. Each transformation was performed in triplicate, and the transformation mixture was plated directly on nylon membranes for the *lacZ* assay.

**Figure 3:**
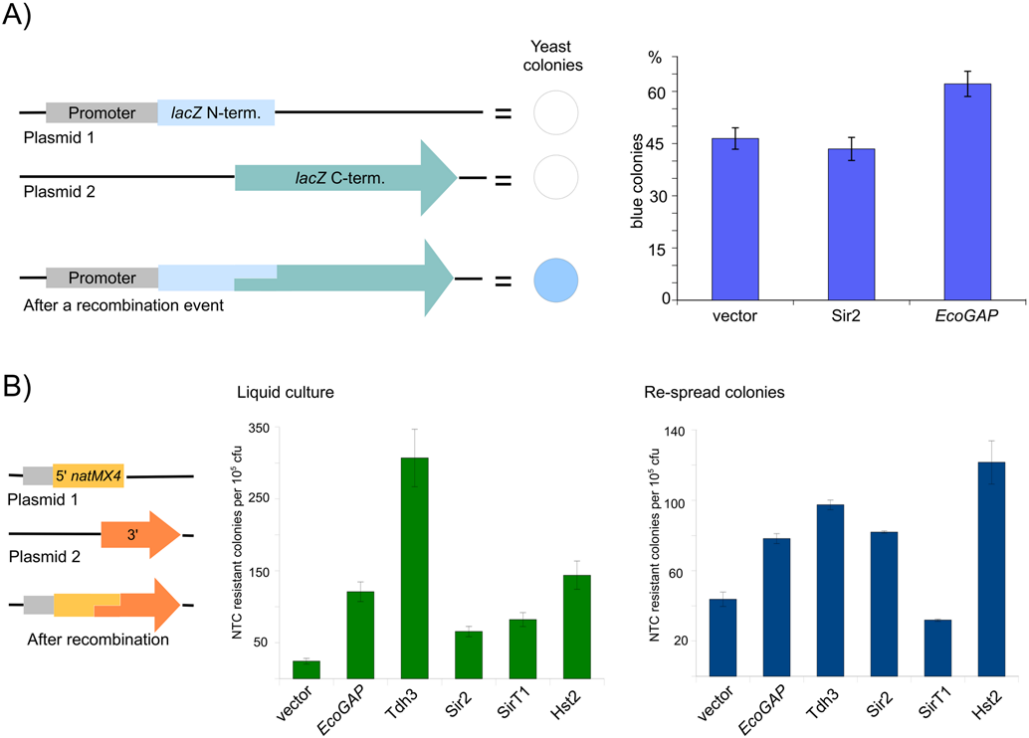
Overexpression of GAPDH paralogues increases plasmid recombination frequency. (A) Principle of the *lacZ*-based plasmid recombination assay (left panel). Plasmid 1 carries a yeast promoter that drives expression of the N-terminal part of ß-galactosidase, and plasmid 2 encodes the C-terminal part. The ß-galactosidase fragments overlap by 564 bp. When the plasmids recombine, a functional *lacZ* expression vector is reconstituted and yeast colonies turn blue in a membrane-based X-GAL assay. (Right panel) The vectors encoding for both ß-galactosidase fragments were transformed in triplicate with an empty vector, p(*HIS3*)-*SIR2*, or p(*HIS3*)-*Eco*GAP and were plated on nylon membranes. Cells were grown at 30°C for 4 days, assayed for ß-galactosidase, and air-dried in the dark. Blue and white colonies were counted. Error bars indicate the standard deviation. (B) *natMX4*-based recombination assay (left panel). Similar to the *lacZ* assay, two plasmids encoding for overlapping fragments of the *natMX4* marker cassette were transformed into yeast. When the plasmids recombine, a functional *natMX4* cassette is reconstituted, allowing growth on NTC- containing media. Yeast containing 5′ and 3′ *natMX4* plasmids was transformed along with a third plasmid encoding the indicated protein (middle panel). Fifty colonies from each transformation were pooled, grown overnight, and spread on plates in triplicate with or without NTC. The Y axis indicates the number of NTC resistant colonies per 10^5^ cfu. Results are also shown with colonies directly re-spread on the NTC media, without re-growth in liquid media (right panel).

As illustrated in Figure 3A, about 45% of the yeast colonies transformed with the empty *HIS3* vector turned blue, indicating that these colonies contained yeast cells expressing a functional ß-galactosidase protein. The number of blue colonies was very similar in the Sir2-overexpressing cells. Remarkably, the number of blue colonies was greatly increased in the *Eco*GAP-overexpressing cells. These results indicate that GAPDH overexpression increases recombination between yeast plasmids in the wild-type background.

In general, the number of *lacZ* positive colonies was very high, probably because the *lacZ* assay is highly sensitive and only a few ß-galactosidase-expressing cells are required to cause a color shift of the whole colony. To analyze, if the recombination events occurred before or during colony formation, we re-spread colonies on a nylon membrane and tested for ß-galactosidase activity. In no case, all colony descendants were *lacZ* positive, indicating that recombination predominantly occurred during colony formation.

We next set up a similar assay permitting an authentic quantification of plasmid recombination events. We used a *natMX4* marker cassette from the pAG25 plasmid that confers resistance against the antibiotic nourseothricin [35]. As for the *lacZ* assay, we generated two vectors, one containing the 5′ and the other containing the 3′ region of the *natMX4* cassette, with an overlap between the fragments of 160 bp. Both vectors were transformed into BY4741 cells, and double-transformants were selected in SC^-LEU-URA^ media. These transformants were re-grown and transformed with the third plasmid encoding the protein to be studied.

In the first experiment, we pooled 50 individual clones from each transformation, grew them overnight in 50 ml YPD, and plated a dilution series in triplicate on SC^-LEU-URA-HIS^ or YPD containing 75 µg/ml nourseothricin (NTC, Jena Bioscience). As illustrated in Figure 3B (middle panel), we tested several plasmids and counted the number of colonies growing on the NTC-containing media. Cultures containing the empty *HIS3* vector produced the lowest number of NTC-resistant colonies (24 NTC resistant clones per 10^5^ cfu's on SC^-LEU-URA-HIS^ media). The number of NTC-resistant colonies was four times higher in *Eco*GAP expressing cells, and about 10 times higher in Tdh3-expressing cells. Expression of Sir2 and SirT1 increased the number of recombinants as well, but not with the magnitude observed with the GAPDH paralogues. Finally, expression of the Sirtuin paralogue Hst2 had a stronger effect on the plasmid recombination rate than its homologues, but was still weaker than yeast GAPDH Tdh3. These results confirm that overexpression of GAPDH paralogues significantly affect the plasmid recombination rate.

Interestingly, the results differed slightly from those obtained from the *lacZ*-based assay. Sir2 had no effect in the *lacZ* assay, but slightly increased the number of NTC-resistant recombinants in the *natMX4* assay. The two assays differ in two ways: first, transformants are exposed to positive growth selection in antibiotic-containing media in the *natMX4* assay, but not in the *lacZ* assay. Second, the clones in the *natMX4* assay were grown in liquid culture, whereas the *lacZ* assay was performed with plate-grown colonies.

To determine whether these differences could account for the observed phenotypes, we modified our *natMX4* assay by spreading the clones on the NTC-containing media without re-growing them in liquid media. As illustrated in Figure 3B (right panel), the number of NTC-positive clones carrying empty vector and overexpressing Sir2 or Hst2 was very similar in both experiments, indicating that the different growth conditions did not significantly affect the recombination rate in these transformants. In contrast, SirT1 produced more NTC-resistant clones compared to the empty vector in the liquid-growth experiment, but showed a slightly lower number of clones in the plating assay.

Interestingly, the growth conditions had a major effect on cells overexpressing GAPDH. *Eco*GAP increased the recombination frequency by a factor of four in the liquid culture assay, whereas the recombination frequency was increased by about two-fold in the direct-plating assay. This phenomenon was even stronger with yeast GAPDH: Tdh3 overexpression caused a 10-fold higher rate of recombination in the liquid culture, but only a three-fold higher rate in the direct-plating assay.

The differences between the two conditions might be surprising, but are reasonable. Glycolysis is responsible for fermentative energy production during the exponential growth phase, and since GAPDH is a major regulator of this pathway, it makes sense that its influence varies with the rate of glycolytic activity.

### Conclusions

Sir2 plays an essential role in gene silencing and in maintaining chromatin structure. Null mutants of Sir2 are deficient in heterochromatin formation and have defects in chromatin structure. Extra copies of Sir2 have been shown to increase the stability of yeast rDNA cycles and to extend the average and maximum lifespan of this single-cell eukaryote [22]–[24], [36].

Sir2 has previously been associated with plasmid stability. Silencing mediated by the 2 µ autonomous replicative sequence is Sir2-dependent [37], and Sir2 null mutants accumulate negative plasmid supercoils. Overexpression of Sir2 leads to positively supercoiled plasmid topoisomers, an effect that depends on Histone 4-K16 acetylation and the Sir2 expression level [38]. It is therefore likely that an increase in Sir2 activity destabilizes episomes and therefore triggers homologous recombination between plasmids.

Here, we show that yeast cells with deregulated glycolysis exhibit an elevation in plasmid recombination rate that is independent of plasmid origin (both 2 µ and centromere-containing plasmids were affected). This increased plasmid recombination rate occurs both in GAPDH-deficient yeast cells overexpressing Sir2 and in wild-type yeast cells overexpressing GAPDH. Although GAPDH is a multifunctional protein, this effect seems to depend on its glycolytic function, since modulating the activity of Tpi, which simulates the metabolic consequences of GAPDH inactivation [14], has a strong effect on the phenotype.

The effect of Sir2 on chromatin structure depends on its enzymatic activity as a protein deacetylase. Sir2 requires oxidized NAD^+^ for forming O-actyl-ADP-ribose by transferring an acetyl group to its ADP-ribose part [28], [39]. Consequently, Sir2 depends on the glycolytic redox cofactor, a fact that represents a direct link between carbohydrate catalysis and chromatin silencing.

**Figure 4:**
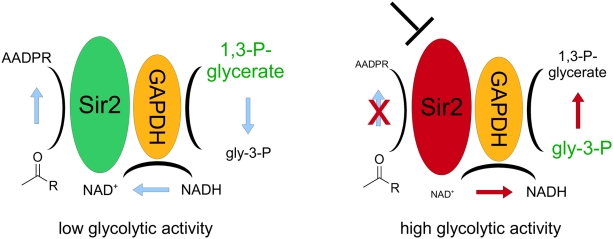
Metabolic interplay of GAPDH and Sir2. GAPDH catalyzes the oxidation of NADH: it generates NAD^+^ in close proximity to Sir2. Under conditions with high glycolytic flux, higher amounts of glyceraldehyde-3-phosphate (gly-3-P) are produced, driving the backward reaction. In this case, GAPDH reduces more NAD^+^ to NADH and inhibits the deacetylation reaction by depleting NAD^+^; NAD^+^ is a limiting factor for the Sirtuins to catalyze the formation of O-acetyl-ADP-ribose (AADPR) via the transfer of acetyl groups.

Remarkably, the nicotinamide concentration has been shown to be the limiting factor for *Sir2* activity [40]. Moreover, Sir2 and GAPDH are present in the same protein complex [25]. Indeed, these facts propose a mechanism for the interplay of GAPDH and Sir2 (Figure 4): In close proximity to Sir2, GAPDH can provide the required NAD^+^ by oxidizing NADH. However, this is dependent on the 1,3-bisphosphoglycerate/glyceraldehyde-3-phosphate ratio. In case of high glycolytic activity, glyceraldehyde-3-phosphate is produced at much higher rates, enforcing the reverse reaction: GAPDH would metabolize NAD^+^ rather than providing it.

Thus, under conditions of low glycolytic activity, GAPDH could enhance Sir2 activity, but under conditions with high glycolytic activity, the opposite is expected to occur. Consequently, dependent on the metabolic activity, GAPDH might be able to both, activate and inhibit Sir2.

This finding is consistent with earlier observations that link Sir2 and glycolysis with chromatin structure: reducing the glucose concentration in the media from 2% to 0.5% dramatically extends the replicative lifespan of *S. cerevisiae*. Lin and colleagues reported that cells deleted for Sir2 lack this phenotype [40], [41]. Conversely, extra copies of Sir2 extended the replicative lifespan in full glucose media [36].

An interesting future question regards the biological role of the very close relation of carbohydrate metabolism and recombination. One might speculate that under strong nutrient deprivation and stress conditions (both influencing the glycolytic flux) a higher rate of recombination increases the genetic diversity of a given yeast population, thus, facilitating the evolutionary adoption to a changing environment [42]. Supportingly, earlier studies also observed a correlation between yeast's nutritional states and meiotic recombination [43]. Of course, much more work is required to fully understand how glycolysis affects cellular processes and what the direct and indirect targets of Sir2 are. Our findings, as well as the recombination assay presented here, provide a good platform for answering these questions.

## Materials and Methods

### Yeast growth conditions

Yeast was grown in yeast peptone dextrose (YPD) or synthetic complete (SC) media containing 2% glucose as a carbon source. *URA3* counter-selection was performed on SC media containing 0.15% fluoroorotic acid (5′FOA, Fluorochem, UK). Cells carrying the *natMX4* marker were grown on YPD containing 75 µg/ml nourseothricin (Jena Bioscience, Germany).

### Plasmid generation

Plasmids were generated by classic restriction/ligation procedures and are listed in Table 1. The vector backbones p423GPD, p416GPD, p413TEF and p413GPD were described by [44], and pRS425 by [45]. Human Rpi1 and SirT1 coding sequences were amplified from a human fetal cDNA library (Clontech), mouse SirT1 from a mouse testis cDNA library (Clontech), *K. lactis GDP1* from p1696 [46], *SIR2* from pAR14 [47], Hst2 from BY4741-, and *Eco*GAP and *lac*Z from *E. coli* genomic DNA (strains Xl1blue and GM2929, respectively). Sir2 mutants, Sir2^H364Y^ and Sir2^P394L^, were generated by site-directed PCR mutagenesis; all cloning experiments involving a PCR were verified by sequencing.

**Table 1:** Plasmids used in this study.

Name	Vector backbone	*S.c.* origin	Aux.	*S.c.* prom	Encoded prot. (species)	Cloning sites	Reference
p(*HIS3*)-EcoGAP	p423GPD	2 µ	*HIS3*	*GPD1*	*Eco*GAP (*E. coli*)	*Bam*HI/*Xho*I	[14]
p(*URA3*)-EcoGAP	p423GPD	2 µ	*URA3*	*GPD1*	*Eco*GAP (*E. coli*)	*Bam*HI/*Xho*I	This study
p(*URA3*)-*RPI*	p416GPD	cen	*URA3*	*GPD1*	Rpi1 (*H. sapiens*)	*Bam*HI/*Xho*I	This study
p(*HIS3*)-*RPI*	p413GPD	cen	*HIS3*	*GPD1*	Rpi1 (*H. sapiens*)	*Bam*HI/*Xho*I	This study
p(*URA3*)-human *TPI1*	p416GPD	cen	*URA3*	*GPD1*	Tpi1 (*H. sapiens*)	*Bam*HI/*Xho*I	[31]
p(*URA3*)-yeast *TPI1*	p416GPD	cen	*URA3*	*GPD1*	Tpi1 (*S. cerevisiae*)	*Bam*HI/*Xho*I	[31]
p(*HIS3*)-*TDH3*	p423GPD	2 µ	*HIS3*	*GPD1*	Tdh3 (*S. cerevisiae*)	*Bam*HI/*Xho*I	[14]
p(*URA3*)-*TDH3*	p426GPD	2 µ	*URA3*	*GPD1*	Tdh3 (*S. cerevisiae*)	*Bam*HI/*Xho*I	This study
p(*HIS3*)-*SIR2*	p413TEF	cen	*HIS3*	*TEF1*	Sir2 (*S. cerevisiae*)	*Eco*RI*/Sal*I	This study
p(*HIS3*)-*SIR2* ^H364Y^	p413TEF	cen	*HIS3*	*TEF1*	Sir2^H364Y^ (*S. cerevisiae*)	*Eco*RI*/Sal*I	This study
p(*HIS3*)-*SIR2* ^P394L^	p413TEF	cen	*HIS3*	*TEF1*	Sir2^P394L^ (*S. cerevisiae*)	*Eco*RI*/Sal*I	This study
p(*HIS3*)-SirT1	p413TEF	cen	*HIS3*	*TEF1*	SirT1 (*H. sapiens*)	*Eco*RI*/Sal*I	This study
p(*HIS3*)-SirT2	p413TEF	cen	*HIS3*	*TEF1*	SirT2 (*H. sapiens*)	*Eco*RI*/Sal*I	This study
p(*HIS3*)-SirT2^mm^	p413TEF	cen	*HIS3*	*TEF1*	SirT2 (*M. musculus*)	*Eco*RI*/Sal*I	This study
p(*HIS3*)-*HST2*	p413TEF	cen	*HIS3*	*TEF1*	Hst2 (*S. cerevisiae*)	*Eco*RI*/Sal*I	This study
p(*HIS3*)-*GDP1*	p413GPD	cen	*HIS3*	*GPD1*	Gdp1 *(K. lactis*)	*Bam*HI/*Xho*I	This study
p426GPD-*lacZ*-NT	p426GPD	2 µ	*URA3*	*GPD1*	5′ region of *lac*Z bp 1-843 (*E. coli EG10527*)	*Bam*HI/*Cla*I	This study
pRS425-*lacZ*-CT	pRS425	2 µ	*LEU2*	*none*	3′ region of *lac*Z bp 280-3075 (*E. coli EG10527*)	*Bam*HI/*Xho*I	This study
p426GPD-*natMX4*-5′	p426GPD	2 µ	*URA3*	*internal(TEF1)*	5′ region of *natMX4* from pAG25	*Sac*I*/Sal*I	This study
pRS425-*natMX4*-3′	pRS425	2 µ	*LEU2*	*none*	3 region of *natMX4* from pAG25	*Sac*I*/Sal*I	This study

### Yeast strain generation

The haploid *MAT*a strain BY4741 [48] was used as the basis for all experiments. The Δ*tdh1*Δ*tdh2*Δ*tdh3 strain* (MR173) was generated using the BY4741-based Δ*tdh3* strain (*tdh3*Δ::*kanMX4*), which was produced by the yeast gene deletion consortium. The strain was transformed with the plasmid p(*URA3*)-*EcoGAP*; subsequently, *TDH2 (tdh2*Δ*::MET15*) and *TDH1* (*tdh1*Δ*::LEU2*) were deleted by homologous recombination. The Δ*tdh1*Δ*tdh2*Δ*tdh3* Δ*zwf1* strain was generated by deleting *ZWF1* in MR173 by replacing the gene with *natMX4*. The Δ*rki1* strain was generated by transforming BY4741 with the plasmid p(*URA3*)-*RPI* and subsequent depletion of the *RKI1* gene by *MET15*. Yeast strains in which *Tpi1* was deleted (MR101, MR105 and MR110) were described previously [14], [31].

### Plasmid recombination assays

#### *lacZ*-based recombination assay

First, the yeast strain BY4741 was transformed with the plasmids p426GPD-*lacZ*-NT and pRS425-*lacZ*-CT. Then, respective transformants were selected on SC^-LEU-URA^ plates, re-grown in liquid SC^-LEU-URA^ media, and transformed in triplicate with the *HIS3* plasmids to be studied. The transformation mixture was plated directly on nylon membranes (“Magna Charge” nylon transfer membrane, Micron Separation, USA) placed on SC^–URA-LEU-HIS^ agar and incubated at 30°C until yeast colonies were grown. Then, the membranes were detached from the agar, shock-frozen in liquid nitrogen, and placed on Whatman paper saturated with buffer (60 mM Na_2_HPO_4_, 40 mM NaH_2_PO_4_, 10 mM KCl, 1 mM MgSO_4_, 0.15% X-Gal, and 10 mM DTT, pH = 7.0). After 4–5 hours of incubation at 37°C, membranes were air-dried in the dark before blue and white colonies were counted.

#### *natMX4*-based recombination assay

The *natMX4* based recombination assay was performed in a similar fashion as the *lacZ* assay. Two overlapping fragments of the 5′ and 3′ regions of *natMX4* were amplified from pAG25 [35] and cloned into the *URA3* (p426GPD) and *LEU2* (pRS425) vectors, respectively. Then, yeast cells were transformed with these plasmids and grown on SC^-URA-LEU^ media. Resulting clones were transformed with a third plasmid encoding the protein to be studied, and triple transformants were selected on SC^-URA-LEU-HIS^. Then a) 50 clones from each transformation were pooled and grown overnight in 50 ml YPD and spread on YPD+75 µg/ml NTC (Jena Biotech) and SC^-URA-LEU-HIS^ or b) spread on YPD+NTC and SC^-URA-LEU-HIS^ without re-growth. Colonies were counted after 3 days incubation at 30°C.
